# Molecular Dissection of the Human Ubiquitin C Promoter Reveals Heat Shock Element Architectures with Activating and Repressive Functions

**DOI:** 10.1371/journal.pone.0136882

**Published:** 2015-08-28

**Authors:** Rita Crinelli, Marzia Bianchi, Lucia Radici, Elisa Carloni, Elisa Giacomini, Mauro Magnani

**Affiliations:** Department of Biomolecular Sciences, Section of Biochemistry and Molecular Biology, University of Urbino “*Carlo Bo*”, Urbino, Italy; Lund University, SWEDEN

## Abstract

The promoter of the polyubiquitin *C* gene (*UBC)* contains putative heat shock elements (HSEs) which are thought to mediate *UBC* induction upon stress. However, the mapping and the functional characterization of the *cis*-acting determinants for its up-regulation have not yet been addressed. In this study, the sequence encompassing 916 nucleotides upstream of the transcription start site of the human *UBC* gene has been dissected by *in silico*, *in vitro* and *in vivo* approaches. The information derived from this analysis was used to study the functional role and the interplay of the identified HSEs in mediating the transcriptional activation of the *UBC* gene under conditions of proteotoxic stress, induced by the proteasome inhibitor MG132. Here we demonstrate that at least three HSEs, with different configurations, exist in the *UBC* promoter: two distal, residing within nucleotides -841/-817 and -715/-691, and one proximal to the transcription start site (nt -100/-65). All of them are bound by transcription factors belonging to the heat shock factor (HSF) family, as determined by bandshift, supershift and ChIP analyses. Site-directed mutagenesis of reporter constructs demonstrated that while the distal elements are involved in the up-regulation of *UBC* in response to proteasome inhibition, the proximal one appears rather to function as negative regulator of the stress-induced transcriptional activity. This is the first evidence that an HSE may exert a negative role on the transcription driven by other HSE motifs on the same gene promoter, highlighting a new level of complexity in the regulation of HSFs and in the control of ubiquitin levels.

## Introduction

Ubiquitin (Ub) is a highly conserved polypeptide that is covalently bound to other cellular proteins to signal processes such as protein degradation, protein/protein interaction and protein intracellular trafficking. Since its discovery in the late ‘70s, many research groups have contributed to unravel the complexity and myriad functions of the Ub/proteasome system [1]. However, with the exception of pioneering biochemical studies on the ubiquitin pathway, the mechanisms that regulate the levels of its own components, including ubiquitin itself, have been largely ignored and only recently re-evaluated. Ubiquitin is an abundant protein within cells; it has been estimated that total ubiquitin may represent the 0.26–0.5% in transformed cells lines and the 0.1–0.4% of total proteins in tissues [2–4]. Nevertheless, the Ub content appears to be not redundant and a tight control over total Ub intracellular levels, and its partitioning between the free and conjugated pools exists [5,6]. It is known that levels of monomeric Ub in the cells depend, as for any other protein, on the rate of synthesis and degradation and, for ubiquitin in particular, also on the recycling of Ub moieties from the substrates before they are degraded [7].

As far as it concerns Ub transcription, in mammals it relies on the activity of four genes which encode Ub polyproteins (*UBB* and *UBC*) or fusions between Ub and small ribosomal proteins (*UBA52* and *UBA80*). In all cases, fusion proteins are processed into Ub monomers which are chemically identical [8]. While genes encoding Ub fusions are thought to maintain the basal level of ubiquitin, the polyubiquitin genes appear to have a major role in mediating the cell stress response [9,10]. This idea turned out partially wrong thanks to the observations derived from knockout mice which have clearly highlighted that *UBB* and *UBC* genes constitute an essential source of Ub also under normal conditions [11]. This study has also provided evidence that, at least in mouse fibroblasts, *UBC* is the major contributor in providing extra ubiquitin during stress and that its loss cannot be compensated by induction of the other Ub genes. While the *UBC* gene is not required for activating the heat-shock stress response, it clearly contributes to sustain the response to cellular stress and to thermo-tolerance [11]. Induction of ubiquitin gene transcription during stress is thought to provide an extra source of ubiquitin necessary to remove damaged/unfolded proteins. Indeed, it has been demonstrated that cells expressing mutant ubiquitin become very sensitive to several types of stress, especially when the ubiquitin mutant UbK48R, that interferes with proteolysis of the Ub-protein substrates, is overexpressed [12,13].

The promoter region of the mammalian *UBC* gene has been cloned and several putative consensus sequences for transcription factors have been found [14], including classical heat-shock elements (HSEs) to which stress-induced *UBC* up-regulation has been attributed [15,9]. The HSE is composed of multiple inverted repeats of the nGAAn pentanucleotide which is specifically recognized and bound by a family of transcription factors termed heat shock factors (HSFs). In humans, the HSF family consists of three members, namely HSF1, HSF2 and HSF4 [16]. HSF1 is considered the master regulator of the stress response, however also HSF2 has been shown to participate in the transcriptional regulation of the heat shock response [17].

Despite the fact that Ub is considered a typical heat shock protein [15], a systematic analysis of the *UBC* promoter to map and characterize regulatory sequences containing functional HSEs has not yet been conducted. Furthermore, with the exception of a ChIP-seq analysis that has revealed that both HSF1 and HSF2 occupy the *UBC* promoter in response to heat stress [18], the binding of *trans*-acting factors to the HSEs present in the *UBC* promoter has never been characterized from a functional point of view.

To fulfill this gap of knowledge, in this study, six overlapping DNA fragments (FR1-FR6), encompassing nt −916/+4 of the *UBC* promoter, were used as probes in Electrophoretic Mobility Shift Assays (EMSA). Stress-induced protein factors, binding to FR1 (-916/-759), FR2 (-781/-636) and FR6 (-195/+4) DNA fragments, were detected in nuclear extracts obtained from cells treated with the proteasome inhibitor MG132. By competition experiments, supershift and ChIP analysis, these factors have been demonstrated to belong to the HSF family of transcription factors. Computational analysis retrieved potential HSE nGAAn recognition units in FR1, FR2, FR6 DNA fragments and short synthetic oligonucleotides (ODNs) containing closed nGAAn repeats were analyzed in EMSA for their ability to bind HSFs. The functional role of the *in vitro* validated HSF binding sequences was assessed by site-directed mutagenesis of the *UBC* promoter in luciferase reporter constructs. Three stress-responsive promoter elements were identified: two distal (nt -841/-817 and nt -715/-691) and one proximal (nt -100/-65) to the transcription start site. The distal regions are responsible for the up-regulation of *UBC* transcription upon MG132 treatment, by contrast the proximal one negatively regulates the stress-induced transcriptional activity. To date, modulation of the heat shock response at the transcriptional level has been attributed to the formation of HSF1 and HSF2 homo- and heterotrimers with still unknown mechanism of action [17,19]. Only recently, HSF isoforms with a specific repressor activity have been described [20]. On the other hand, different HSE configurations capable of mediating various degrees of transcriptional activation, have been reported in the literature [21]. To the best of our knowledge, this is the first report of the coexistence of HSEs with activating and repressive functions on the same promoter; these opposing activities could provide a sort of molecular control knob to adjust ubiquitin *de novo* synthesis to the cellular need to remove damaged/unfolded proteins. These results highlight a new regulatory level in the control of ubiquitin expression and in the fine-tuning of the HSF-mediated transcriptional activity.

## Materials and Methods

### Cell culture and treatment

The cervical cancer cell line HeLa was purchased from the American Type Culture Collection (ATCC). Cells were grown in RPMI 1640 medium supplemented with 10% fetal bovine serum, 2 mM glutamine, 100 μg/ml streptomycin and 100 U/ml penicillin at 37°C under 5% CO_2_. Cells were plated in 60 mm dishes at a density of 8x10^5^ cells/dish and incubated 4h with 20 μM MG132 (VWR international s.r.l., Milano, Italy) for nuclear extract preparation and ChIP assay, 10 μM lactacystin (Santa Cruz Biotechnology Inc., Santa Cruz, CA, USA) for ChIP assay. As control, cells were treated with the vehicle alone (DMSO, dimethyl sulphoxide) to a final concentration of 0.04% (v/v). In reporter construct experiments, 48h post-transfection cells were incubated with 20 μM MG132 or DMSO for 8h and then harvested for RNA extraction.

### Electrophoretic mobility shift assay (EMSA)

Nuclear extracts were obtained by low salt/detergent cell lysis followed by high salt extraction of nuclei, as previously described [22]. The sequence and the position of the six primer pairs employed to generate PCR DNA probes (FR1 to FR6) encompassing the cloned promoter sequence were: FR1 (-916) fwd 5’-GAGAAATTTCCATGCCTCCCTGTT-3’ and FR1 (-759) rev 5’-AAAAGAGGCGGAAACCCCACA3’; FR2 (-781) fwd 5’-TGTGTGGGGTTTCCGCCTCT-3’ and FR2 (-636) rev 5’-CGCGGGACAAGGACAATGAC-3’; FR3 (-673) fwd 5’-CGCCCTGAGATCTGCCGAGT-3’ and FR3 (-516) rev 5’-CCTGCGAGATGGACGGGTCT-3’; FR4 (-537) fwd 5’-AAAGACCCGTCCATCTCGCA-3’ and FR4 (-356) rev 5’-CGGCCCGCGTTCCTTAG-3’; FR5 (-371) fwd 5’-TAAGGAACGCGGGCCGCCCA-3’ and FR5 (-162) rev 5’-GTCCTTCTGCTGATACTGGGGTTC-3’; FR6 (-195) fwd 5’-CTCGGCCTTAGAACCCCAGTATC -3’ and FR6 (+4) rev 5’-AACTAGCTGTGCCACACCCG-3'. These probes were amplified using as a template the -916/+878 *UBC* gene sequence, previously cloned into the pGL3-basic vector (Promega s.r.l., Milano, Italia) (P1 construct) [23]. Primers used to generate exon 1/intron probes (probes I to VI) have been previously described [23]. HPLC-purified synthetic ODNs were purchased from Thermo Fisher Scientific GmbH (Ulm, Germany); sequences of wild-type and mutated *UBC* ODNs are depicted in supplementary figures. The ODN containing the canonical HSE consensus sequence (ODN-cHSE), used as specific competitor, was: 5’-CTAGAACGTTCTAGAAGCTTCGAG-3’. Double-stranded oligonucleotides and gel-purified PCR products, were 5’ end-labeled with [γ-^32^P] ATP (Perkin Elmer Life Sciences, Boston, USA) and T4 polynucleotide kinase (T4 PNK, Roche Diagnostics, Mannheim, Germany). Nuclear extracts (5 μg) were preincubated with 3 μg of double-stranded non-specific DNA competitor poly(dI-dC) (Amersham Pharmacia Biotech, Piscataway, USA) for 10 min on ice in binding buffer (20 mM Hepes-KOH, pH 7.9, 0.1 M KCl, 5% (v/v) glycerol, 0.2 mM EGTA, 0.2 mM EDTA, 1 mM dithiothreitol). After this time, a ^32^P-end-labeled DNA probe was added to the mixtures at a final concentration of 4 nM and the incubation was continued for an additional 30 min. Reaction mixtures were then submitted to electrophoretic separation on 5% native polyacrylamide gels (29:1 cross-linked) in Tris-glycine buffer (25 mM Tris base, 192 mM glycine). DNA/protein complexes were detected by exposing the dried gel in a Molecular Imager (Bio-Rad laboratories, Milano, Italy). For competition experiments, nuclear extracts were incubated with a 50-fold excess of a double-stranded competitor ODN for 10 min before adding the ^32^P-labeled probe. For supershift experiments, nuclear extracts were incubated with 1 μg of anti HSF1 (E-4) (sc-17757X) or anti HSF2 (C-20) (sc-8062X) antibodies (Santa Cruz Biotechnology Inc.) for 30 min at room temperature prior to addition of the radiolabeled probe.

### ChIP assay

ChIP assays were performed using the ChIP assay kit (Upstate Biotechnology Inc., New York, NY, USA) essentially as described by the manufacturer. Cross-linked DNA was subjected to twelve 15 s sonication pulses at 45 watts by using a Labsonic 1510 Sonicator (Braun, Melsungen, Germany), to obtain sheared chromatin with an average size of 200/500 bp. For each immunoprecipitation 2x10^6^ cell equivalents of sheared chromatin were incubated overnight at 4°C with 10 μg of anti HSF1 (H-3) (sc-9144X) or anti HSF2 (H-300) (sc-13056X) antibodies (Santa Cruz Biotechnology Inc.) or with no antibody, as control. After decrosslinking and treatment with RNase A and Proteinase K, immunoprecipitated DNA as well as Input DNA (1% of the input extract) were purified with spin columns and quantitatively analyzed by RealTime PCR using the SYBR green RealTime master mix. The following primer sets were used to analyze HSF1 and HSF2 binding to the target regions: FR1 forward and reverse (described above) and FR6 ChIP forward 5’-ACTCGGCCTTAGAACCCCAGTA-3’ and reverse 5’-CTCGCCTGTTCCGCTCTCT-3’ which amplify the *UBC* promoter fragments -916/-759 and -196/-96, respectively. As negative and positive controls, the downstream *UBC* intron region, designed as probe V (+608/+766) [23], and the HSE element present in the human *HSP70* promoter [24], were amplified in parallel with the following primer pairs: probe V forward 5’-AGGGTAGGCTCTCCTGAATCGAC-3’ and reverse 5’-TCACAAAACACACTCGCCAACC-3’; Hsp70 forward 5’-AGCCTCATCGAGCTCGGTGATTG-3’ and reverse 5’-AAGGTAGTGGACTGTCGCAGCAGC-3’. RealTime PCR data were analyzed according to the 2^−ΔCT^ method; input DNA values were used to normalize the values of immunoprecipitated (IP) samples as follow: ΔCt = Ct of IP samples − Ct input, % input = 2^-ΔCt^ [25].

### Reporter constructs, site-directed mutagenesis and transient transfection

The P1 and P3 constructs have been previously described [23]. They consist of 916 and 371 nt upstream of the transcription start site, respectively, the 63-nt exon 1 and the 812-nt unique intron of the *UBC* gene, cloned in the pGL3-basic firefly luciferase reporter vector (Promega). The P1 construct was mutated using the QuikChange Site-Directed Mutagenesis kit (Stratagene, La Jolla, CA, USA) according to the manufacturer’s instructions. In particular, the core sequence of the nGAAn recognition units, demonstrated to be critical for HSF binding *in vitro*, was changed from GAA to CGC, in order to abrogate transcription factor binding to FR1 and FR2 segments altogether (P1 mut FR1-2 construct) or to the FR6 promoter sequence (P1 mut FR6 construct). The P1 mut FR1-2 construct was obtained after three cycles of mutagenesis which have introduced, respectively, the first and then the second substitution in FR1 and finally the 2 substitutions in FR2, using the following primers: FR1 HSFc-mut 1X forward 5’-GAATGTACAGGAAGGTGGAACGCCAGTCTAGAAGGATGTC-3’ and reverse 5’-GACATCCTTCTAGACTGGCGTTCCACCTTCCTGTACATTC-3’; FR1 HSFc-mut 2X forward 5’-GGTGGAACGCCAGTCTACGCGGATGTCGTTCGCTCAGC-3’ and reverse 5’-GCTGAGCGAACGACATCCGCGTAGACTGGCGTTCCACC-3’; FR2 HSFc-mut 2X forward 5’-GCAGTGTCTCCCCTGCGCAGGCGTCGAGCGTTCCCCAGC-3’ and reverse 5’-GCTGGGGAACGCTCGACGCCTGCGCAGGGGAGACACTGC-3’ (nucleotide changes with respect to the wild-type sequence are underlined). For the generation of the P1 mut FR6 construct the following primers were used: FR6 HSFc-mut 1X forward 5’-GCGAGGAAAAGTAGTCCCGCGTCGGCGATTCTGCGG-3’ and reverse 5’-CCGCAGAATCGCCGACGCGGGACTACTTTTCCTCGC-3’. The mutated sequences were verified by automated sequencing, in both directions, using a PE310 Perkin Elmer capillary sequencer. For cell transfection studies, plasmid DNA was propagated in JM109 bacterial strain and purified by EndoFree Plasmid Maxi Kit (Qiagen Inc., Valencia, CA, USA) to remove bacterial endotoxins. Transient transfection of reporter constructs was performed with Effectene reagent (Qiagen), according to the manufacturer’s protocol. The day before transfection, cells were plated at a density of 3.5x10^5^ cell/well in 6-well plate. 400 ng of DNA were added to each well.

### RNA extraction, reverse transcription (RT) and quantitative Real-Time PCR (qPCR)

Total cellular RNA was prepared using the RNeasy Plus Mini kit (Qiagen). To remove any traces of plasmid DNA, total RNA (up to 10 μg) was treated with 2 units of TURBO DNA-free (Ambion, Austin, TX, USA) for 30 min at 37°C, according to the included protocol. TURBO-treated RNA (1 μg) was reverse-transcribed using the SuperScript First-Strand Synthesis System (Invitrogen, Carlsbad, CA, USA), essentially as indicated in the standard protocol. cDNAs were used as templates in SYBR green quantitative RealTime PCR (qPCR) assays, performed with the Hot-Rescue RealTime PCR kit (Diatheva s.r.l., Fano, Italy). PCR reactions were set up in a volume of 25 μl containing 1X Hot-Rescue RealTime Master Mix, 0.2 μM of gene specific primers, 0.625 units of Hot-Rescue DNA polymerase, 5 μl of a fifty-fold dilution of the RNase H-treated cDNA stock. DNA amplifications were carried out in 96-well reaction plates using ABI PRISM 7700 Sequence Detection System platform (Applied Biosystems, Foster City, CA, USA). Each sample was analyzed in triplicate, and multiple blanks were included in each analysis. qPCR primers (obtained from Sigma-Genosys Ltd, Haverhill, UK) were designed using Primer Express version 2.0. Primer sequences, as well as the relative MgCl_2_ were: luciferase (LUC), forward 5’-TGTACACGTTCGTCACATCTCATCT-3’ and reverse 5’ AGTGCAATTGTCTTGTCCCTATCG-3’ (3 mM MgCl_2_, 91 bp); glyceraldehyde-3-phosphate dehydrogenase (GAPDH), forward 5’-TGCACCACCAACTGCTTAG-3’ and reverse 5’-GATGCAGGGATGATGTTC-3’ (2.5 mM MgCl_2_, 176 bp) [26]. Cycle conditions were: 95°C for 10 min followed by 40 cycles of 15 s at 95°C, 15 s at 60°C and 30 s at 72°C. LUC mRNA expression data, normalized to GAPDH were calculated with the 2^−ΔΔCT^ method [27].

### Computational and statistical analysis

Computational analysis of the *UBC* gene in search for putative HSF-binding *cis*-elements was performed with MatInspector [28] (http://www.genomatix.de/matinspector.html), TESS [29] (http://www.cbil.upenn.edu/cgi-bin/tess/tess) and TF Search [30] (http://cbrc.jp/research/db/TFSEARCH-html) software packages. Statistical significance was calculated using SPSS 20.0 (IBM). Analysis of variance (ANOVA) was used to calculate significance. Differences between values were assumed statistically significant at p< 0.05 (*) and very significant at p< 0.01 (**).

## Results

### Identification of the *UBC* promoter regions containing *cis*-acting elements able to recruit HSFs upon MG132-induced proteotoxic stress

**Fig 1:**
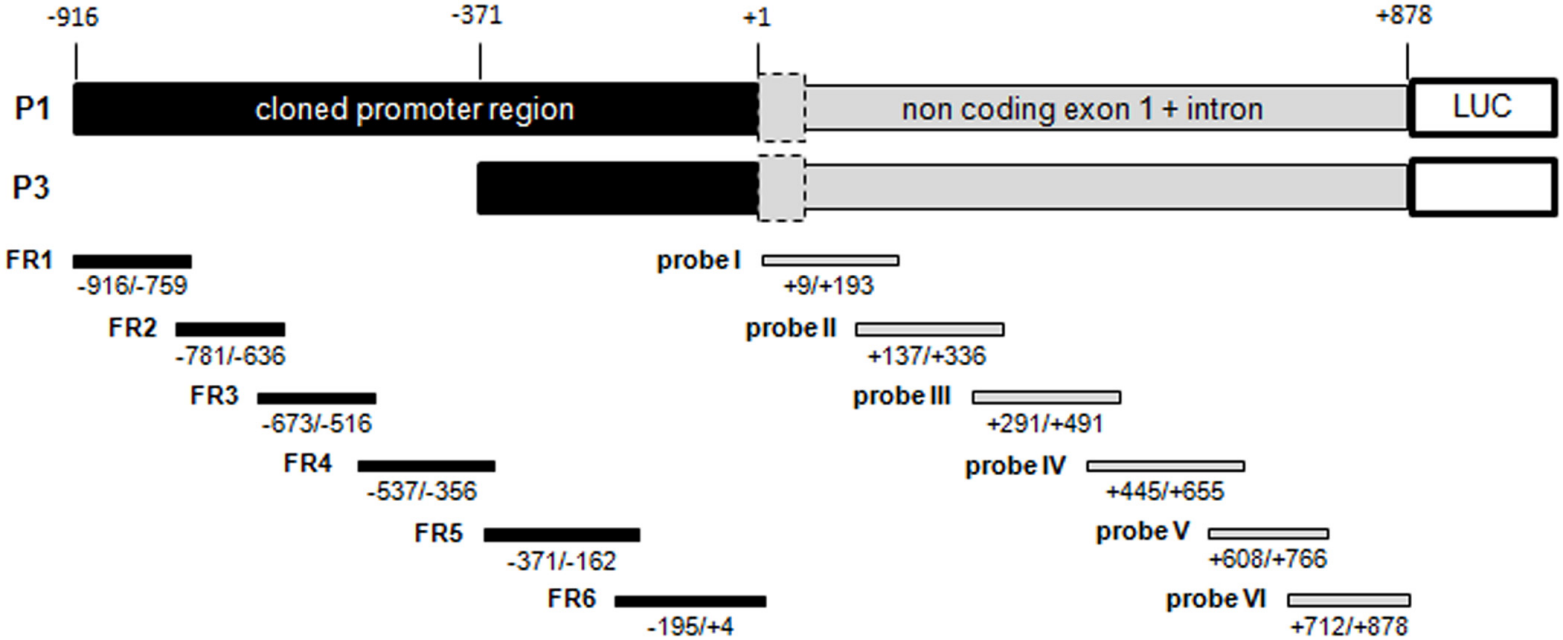
Schematic representation of the cloned *UBC* promoter region. The *UBC* promoter region (black box), the first non coding exon and the unique intron (grey box, the dashed box corresponds to exon 1), as they have been previously cloned into the pGL3-basic firefly reporter vector to generate the constructs named P1 (-916/+878) and P3 (-371/+878) (23). The transcription start site is marked as +1. Depicted to scale are also the PCR-generated DNA fragments used in EMSA. Promoter probes are indicated as FR1-6 (in black); exon/intron probes as probe I-VI (in grey). Position of the DNA segments is indicated. LUC, luciferase.

**Fig 2:**
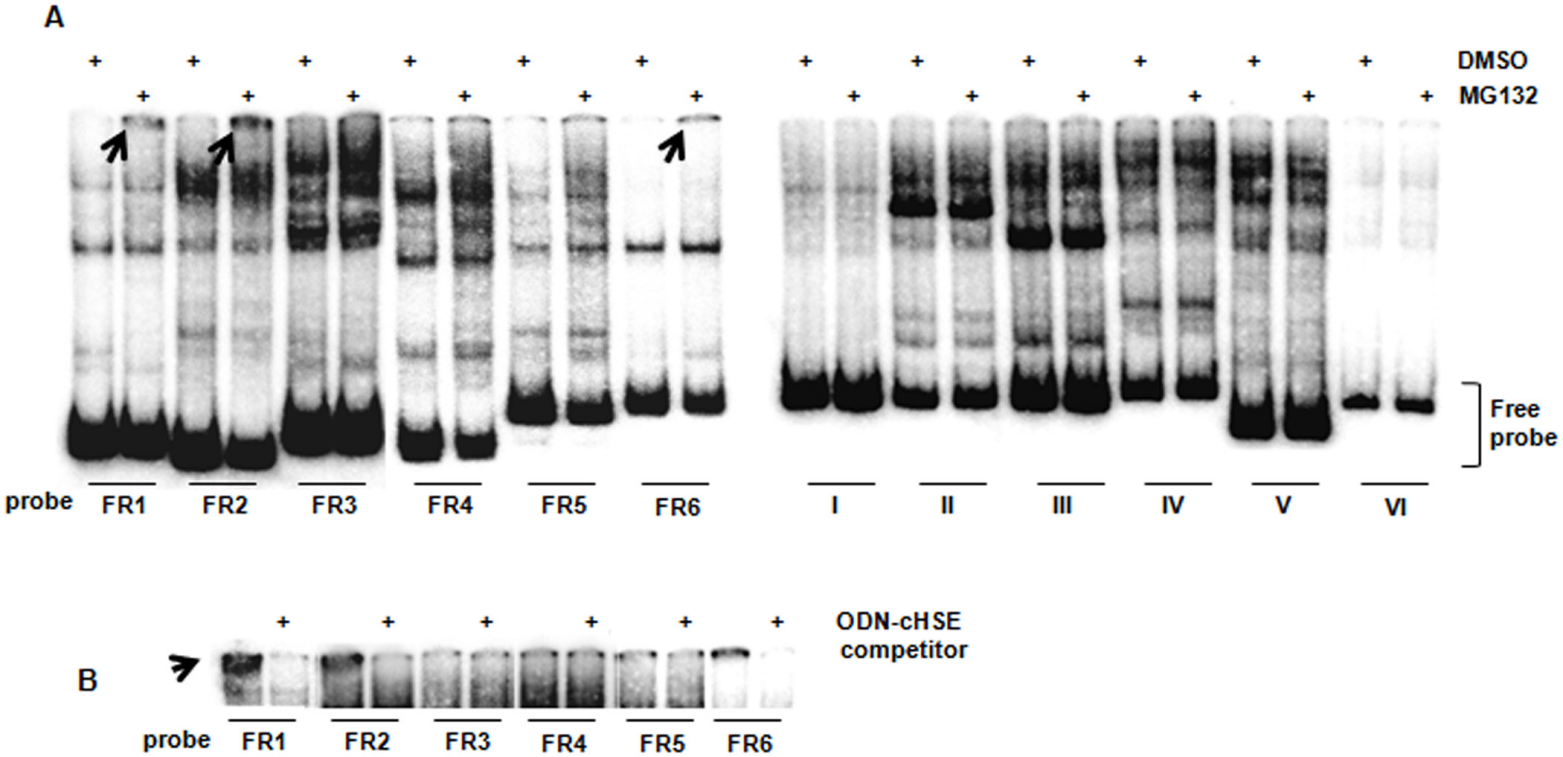
EMSA analysis of the *UBC* DNA fragments generated by PCR. (A) Protein/DNA complexes detected upon incubation of promoter/intron probes with nuclear proteins derived from DMSO- or MG132-treated cells (20 μM, 4h). Nuclear extracts (5 μg) were submitted to EMSA using as probes the twelve PCR-generated DNA fragments encompassing the cloned *UBC* promoter sequence (FR1-6), the first exon and the unique intron (I-VI). Protein-DNA complexes were separated on 5% polyacrylamide gels and the radiographic signal was detected in a GS-250 Molecular Imager. Arrows indicate the position of complexes specifically formed upon MG132 treatment (B) Specificity of MG132-induced protein/DNA complexes was assessed by competition experiments where nuclear extracts from MG132-treated cells were directly incubated with the labeled probes (FR1 to FR6) or pre-incubated with a 50-fold excess of an ODN containing the canonical HSF consensus sequence (ODN-cHSE), before probe addition. The image was cropped to show only the gel region of interest.

To identify the *UBC* promoter regions containing HSEs, six DNA fragments (FR1 to FR6) were designed in order to produce a series of EMSA probes, with overlapping edges, covering 916 nt upstream of the transcription start site (Fig 1, black blocks). The probes were PCR-generated with specific primers, using as a template the P1 reporter vector which has been previously constructed and characterized in our laboratory [23]. HeLa cells were incubated with 20 μM MG132 for 4h; at this time point, the signal intensity in EMSA was still high, while declined at later time points, with a peak of HSF activation between 2 and 4h from the administration of the molecule (S1 Fig). As control, cells were treated with the vehicle (i.e. 0.04% DMSO). Incubation of the ^32^P-labeled DNA duplexes with nuclear extracts deriving from DMSO-treated cells resulted in the appearance of several protein/DNA complexes in bandshift assays (Fig 2A, left panel). The radiographic signals were mostly abrogated upon competition with ODNs containing the Sp1 and/or YY1 (Yin Yang 1) wild-type consensus sequence, but not the mutated one, indicating that these two transcription factors bind *in vitro* to the *UBC* promoter (S2 Fig). The pattern did not change when nuclear extracts were obtained from cells incubated with the proteasome inhibitor MG132 (Fig 2A, left panel). The only exception was represented by very high molecular weight protein/DNA complexes which barely entered the gel and which were observed when the DNA fragments FR1, FR2 and FR6 were used as probe (Fig 2A, indicated by arrows). A similar, although less marked, radiographic signal was also present at the top of the gel with probes FR4 and FR5. In this case, however, it was already evident in the DMSO control (Fig 2A). In light of these results, we reasoned that the very low electrophoretic mobility of the observed complexes could be the consequence of the size of the probes used (from 146 to 210 bp) together with the fact that proteasome inhibitors are known to activate HSFs which bind DNA as trimers. Thus, the specificity of complex formation was further assessed by competition experiments where nuclear extracts from MG132-treated cells were pre-incubated with an excess of an unlabeled ODN containing the canonical HSF consensus sequence (ODN-cHSE), before addition of the radiolabeled probes. As shown in Fig 2B, the competitor was able to interfere with the formation of the MG132-induced protein/DNA complexes in extracts incubated with FR1, FR2 and FR6, strongly suggesting that HSFs are indeed the factors bound to these DNA fragments. The signal for FR5 was only slightly reduced, while no competition was observed with all the other DNA segments, indicating that it is not the result of a specific interaction between HSFs and the labeled probes (Fig 2B). In support to this evidence, none of the software packages used for computational analysis found putative HSEs in FR3, FR4 and FR5.

We have demonstrated that the intron is crucial for the basal transcriptional activity of the *UBC* gene, acting as repository of transcription factor binding sites [31]. Taking advantage of a series of probes (probes I to VI, Fig 1, grey blocks), previously designed to encompass the first untranslated exon and the unique *UBC* intron [23], the same approach, as described above, was used to assess the potential role of these regions in the stress response. No quantitative and/or qualitative changes were observed in the bandshift pattern obtained using nuclear extracts from DMSO- and MG132-treated cells, with all the probes tested (Fig 2A, right panel), allowing to exclude the intron participation to the stress-induced *UBC* up-regulation, at least by binding protein factors.

**Fig 3:**
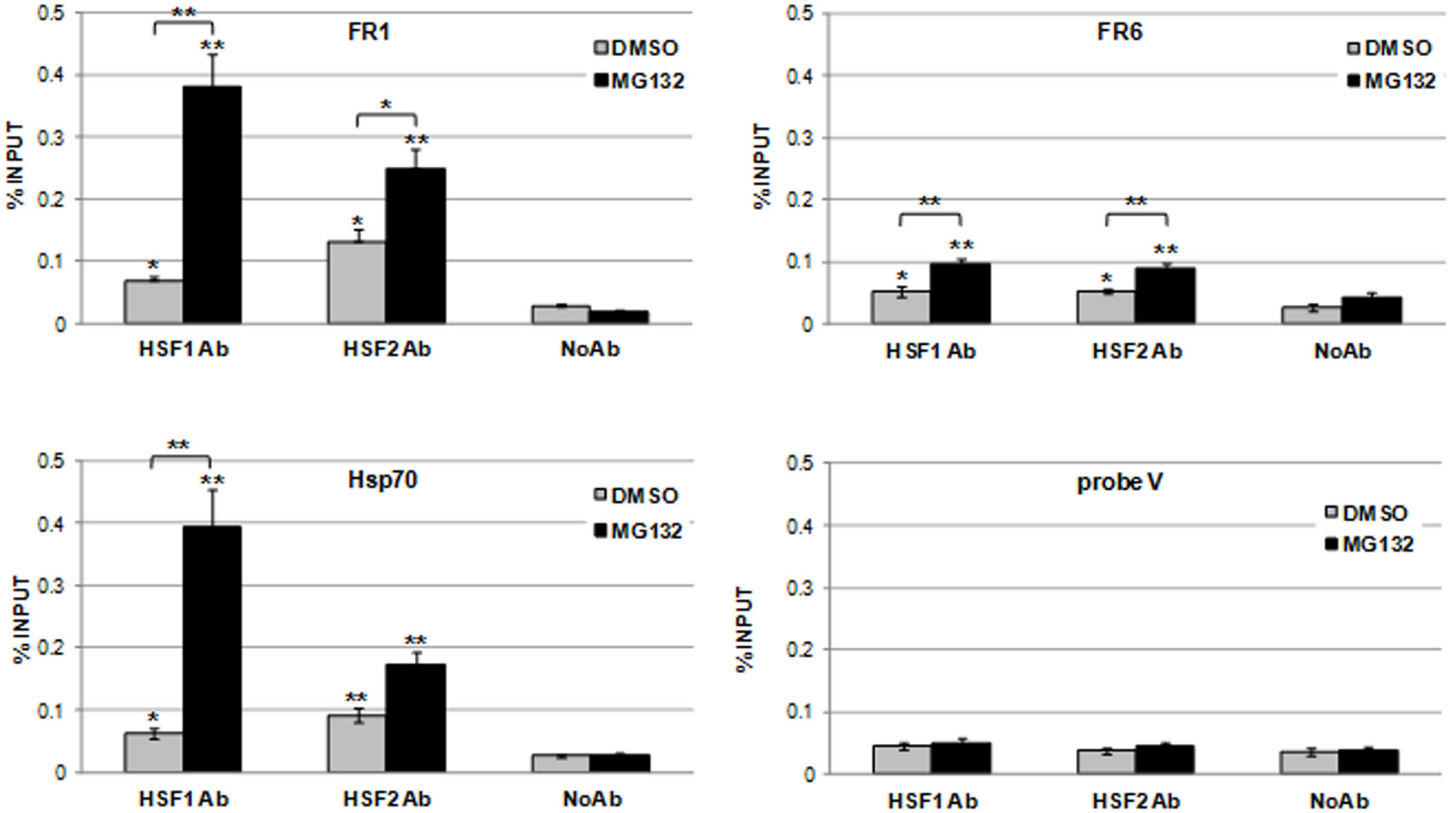
Binding of HSFs to FR1 and FR6 *UBC* DNA regions *in vivo*. ChIP analysis was performed on DMSO- and MG132-treated cells (20 μM, 4h) using specific antibodies against HSF1 and HSF2. Chromatin submitted to the immunoprecipitation procedure in the absence of added antibody (No Ab) was used as internal IP control. RealTime PCR was carried out on chromatin before (input) and after immunoprecipitation using specific primers which amplify the *UBC* promoter regions FR1 (-916/-759) and FR6 (-196/-96). Primers flanking the HSE contained in the human *HSP70* promoter or amplifying the *UBC* intron probe V (+608/+766) were used to generate HSF positive and negative ChIP controls, respectively. Each sample was tested in triplicate. Data were analyzed according to the 2^−ΔCT^ method, normalized to the input DNA and expressed as % input. The average value ± SE was calculated from six independent ChIP analyses. Asterisks indicate statistical significance versus the corresponding No Ab, unless otherwise highlighted in the graphs. Statistical analysis was performed with two-way ANOVA. *p<0.05; **p<0.01.

Having determined the regions containing the *cis*-acting elements able to recruit HSFs *in vitro*, we next investigated whether these factors bind to these *UBC* DNA fragments also in intact cells. To this purpose, we performed ChIP experiments with antibodies against HSF1 and HSF2. Indeed, proteasome inhibition is known to induce the stabilization/activation of HSF2 [32]. Since FR1 and FR2 segments are too closed to be resolved by ChIP approaches, analyses were conducted considering the FR1 segment as target for both regions. Thus, the recruitment of HSFs to the *UBC* promoter *in vivo* was studied by using two primer sets which amplify the FR1 and FR6 segments, respectively. The binding of HSF1 and HSF2 to FR1 and FR6 was already observed in DMSO-treated cells, as demonstrated by a statistically significant enrichment of the corresponding amplification products in samples immunoprecipitated with both anti HSF1 and HSF2 antibodies, compared to chromatin where no antibody was added (noAb) (Fig 3). In response to MG132 treatment, the binding of HSFs further increased, particularly that of HSF1 to region FR1 (5.6-fold vs control). The interaction of HSF2 with FR1 and that of HSF1 and HSF2 with FR6 was almost doubled and the increase over DMSO-treated cells was statistically significant (Fig 3). An HSF layout similar to that of FR1 was detected on the *HSP70* promoter, used as positive ChIP control, with the only exception that the binding of HSF2 upon MG132 administration did not result significantly increased over the basal level (DMSO-treated cells) (Fig 3). By contrast, no enrichment with respect to the noAb sample was detected when intron probe V was amplified (Fig 3), in agreement with the evidence that the *UBC* intron does not bind HSFs (Fig 2A, right panel). A similar, albeit lower, HSF occupancy was found at the FR1 and FR6 *UBC* promoter regions, as well as at the *Hsp70* promoter, in cells treated with the proteasome inhibitor Lactacystin (S3 Fig).

### *In vitro* identification of the nGAAn recognition units critical for HSF binding to the *UBC* promoter

**Fig 4:**
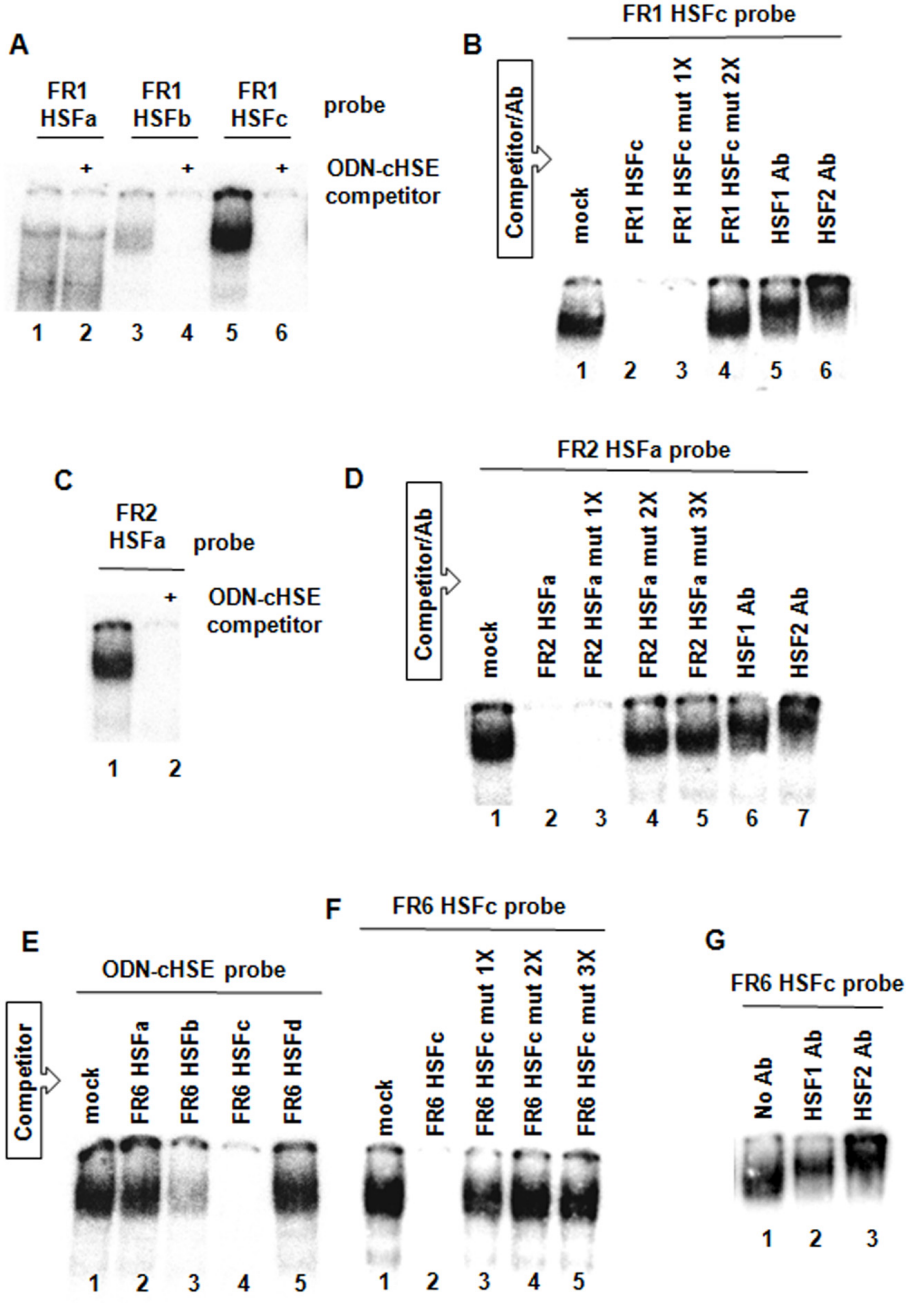
Dissection of the *UBC* FR1, FR2 and FR6 segments. Wild-type and mutant ODNs containing the putative *UBC* HSEs were submitted to EMSA, competitive EMSA and supershift analysis. Nuclear extracts from MG132-treated cells were directly incubated with the indicated ^32^P-ODN probe (lanes 1A, 3A, 5A, 1B, 1C, 1D, 1E, 1F and 1G) or pre-incubated with a 50-fold excess of unlabeled competitor oligonucleotide (lanes 2A, 4A, 6A, 2-4B, 2C, 2-5D, 2-5E and 2-5F) or antibodies against HSF1 and HSF2 (lanes 5-6B, 6-7D and 2-3G), before addition of the radiolabeled probe. ODN-cHSE: ODN containing the classical HSE consensus sequence. The images were cropped to show only the gel region of interest.

To circumscribe the HSF binding sequences within FR1, FR2 and FR6, the *UBC* segments were submitted to sequence analysis with “TF search”, “MatInspector” and “TESS” in search of putative HSF binding sites. All the software packages retrieved sequences containing potential HSE nGAAn recognition units which, unfortunately, were not located in the context of a typical HSE motif, consisting of at least three inverted repeats of the pentameric nGAAn motif [16]. Nonetheless, all the matches obtained with the three programs were considered, including those that were not concordant; position and orientation of the core sequence GAA are reported in S4 Fig for each DNA segment. Based on this analysis, short synthetic ODNs, at least 25 bp-long, were designed in order to include two or more closed nGAAn units, whenever possible, and some of the adjacent nucleotides, without extending the length of the ODNs over the 35/40 bp. Indeed, it has been demonstrated that two repetitions of the pentameric motif are sufficient for HSF binding *in vitro* [33]. The oligonucleotides were used in EMSA as probes to assess their effective ability to recruit HSFs. Three ODNs were selected on FR1 and termed FR1 HSFa, FR1 HSFb, FR1 HSFc (S4 Fig). The ODNs were labeled with ^32^P and incubated with a nuclear extract obtained from MG132-treated cells. The formation of protein/DNA complexes in bandshift assay was observed with all of them (Fig 4A, lanes 1, 3, 5), but the signal was specifically abrogated by an unlabeled competitor ODN, containing the canonical HSE sequence (ODN-cHSE), only in the case of FR1 HSFb and FR1 HSFc (Fig 4A, lanes 4, 6). Notably, the radiographic signal corresponding to the DNA/protein complex obtained with FR1 HSFc was more marked than that produced by the FR1 HSFb probe. Since the two probes had the same specific activity, this indicates that FR1 HSFc has an apparent greater binding affinity for HSFs than FR1 HSFb (Fig 4A, lanes 3, 5). Based on this result, FR1 HSFc was further characterized by mutagenesis and supershift assay in order to identify the nGAAn units critical for binding and demonstrate the specific binding of heat shock factor proteins. A single complex was identified in extracts incubated with FR1 HSFc in the absence of competitor (Fig 4B, lane 1), the signal was up-shifted by anti HSF1 and HSF2 antibodies (Fig 4B, lanes 5, 6). Complex formation was abrogated by competition with the same unlabeled ODN (Fig 4B, lane 2) and with an FR1 HSFc ODN mutated in the first putative nGAAn unit (Fig 4B, lane 3 and S5 Fig). No competition was observed with an ODN competitor where both nGAAn units were mutated, indicating that only unit 2 or both units are critical for binding (Fig 4B, lane 4 and S5 Fig). On the other hand, it is worth noting that mutation of just one nGAAn block within an HSE, typically containing three or more blocks, could not be indeed sufficient to abrogate *in vitro* HSF binding since HSF/DNA interactions can still occur on incomplete HSEs carrying two contiguous repeats of the pentameric motif [33]. Thus, if the introduction of one block mutation is ineffective in abrogating HSF binding *in vitro* (i.e. unit 1 in FR1 HSFc), it cannot be concluded that the block is not important for HSF binding *in vivo* where HSFs trimers need to contact three pentameric units for efficient DNA binding.

An approach similar to that described for FR1 was used for FR2, where it was possible to include all the putative nGAAn pentanucleotides in one ODN termed FR2 HSFa (S4 Fig). FR2 HSFa formed a complex with nuclear proteins derived from MG132-treated cells (Fig 4C and 4D, lane 1); the complex was displaced by addition of an excess of unlabeled ODN-cHSE (Fig 4C, lane 2) and supershifted by antibodies against HSF1 and HSF2 (Fig 4D, lanes 6, 7). Complex formation was abrogated by competition with an FR2 HSFa ODN wild-type (Fig 4D, lane 2) and mutated in the nGAAn unit 2 (Fig 4D, lane 3 and S5 Fig), but not by an oligonucleotide where two (units 1 and 2) or all the three pentameric motifs were mutated (Fig 4D, lanes 4, 5 and S5 Fig). Also in this case it was not possible to establish whether only the first or the first and the second nGAAn motifs are necessary and sufficient to abrogate HSF binding for the reasons explained above. Slightly different was the approach used to analyze the DNA segment FR6 where it was necessary to design at least 4 ODNs termed FR6 HSFa, FR6 HSFb, FR6 HSFc, FR6 HSFd (S4 Fig). Due to the high number of probes to test in EMSA, the ODN-cHSE was labeled and used as probe in bandshift experiments where the different FR6 ODNs were the competitors (Fig 4E). Complete abrogation of HSFs/ODN-cHSE complex formation was obtained with FR6 HSFc (Fig 4E, lane 4), while FR6 HSFb only partially competed out the labeled probe (Fig 4E, lane 3). No competition was observed with the other ODNs (Fig 4E, lanes 2, 5). FR6 HSFc was selected for further analyses where the ODN was ^32^P-labeled and used as probe in competition and supershift experiments. Incubation of FR6 HSFc with nuclear extracts from MG132-treated cells led to the formation of a single shifted radiographic signal (Fig 4F and 4G, lane 1), which disappears upon competition with wild-type FR6 HSFc (Fig 4F, lane 2), but not with an FR6 HSFc carrying mutations in nGAAn unit 2 (FR6 HSFc mut 1X), 2 and 3 (FR6 HSFc mut 2X), or 1, 2 and 3 (FR6 HSFc mut 3X) (Fig 4F, lanes 3–5 and S5 Fig). Thus, in this case, mutation of recognition unit 2 was already sufficient to abrogate transcription factor binding. The protein/DNA complex was supershifted by antibodies against both HSF1 and HSF2, demonstrating the specific binding of HSFs to this *UBC* sequence (Fig 4G, lanes 2, 3). On the whole, these evidences allowed us to circumscribe the *UBC* HSF binding regions within FR1 nt -841/-817, FR2 nt -715/-691 and FR6 nt -100/-65 and to identify in each of them the minimal sequence changes sufficient to abrogate HSF binding.

### Functional characterization of the identified stress-responsive elements

**Fig 5:**
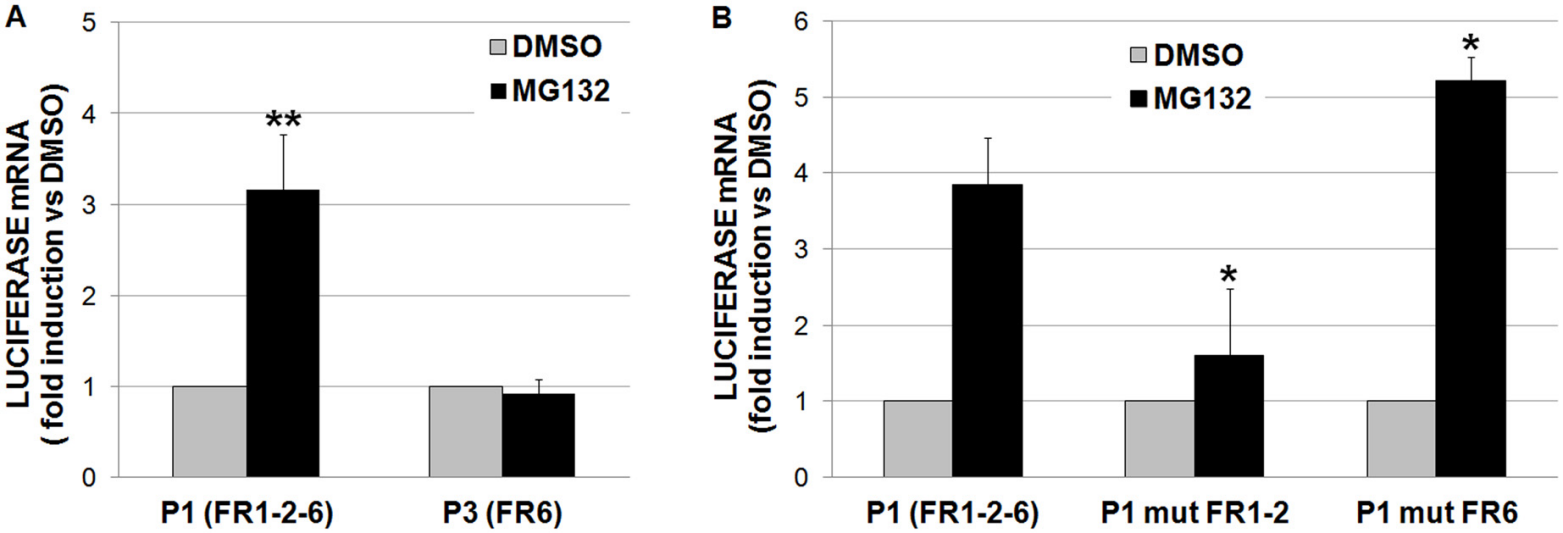
Reporter construct gene expression analysis. (A) The *UBC* promoter-pGL3 constructs P1 (-916/+878) and P3 (-371/+878) containing FR1-2-6 and only FR6, respectively, were transfected in HeLa cells. Fourty-eight hours post-transfection, cells were treated with the DMSO vehicle or MG132 (20 μM). After 8h, luciferase mRNA levels were determined by RealTime PCR. Expression data, normalized to the housekeeping GAPDH gene, were analyzed by the 2^-ΔΔCT^ method and referred to the value obtained in DMSO-treated cells. The data represent the mean fold induction ± S.E. of 5 independent experiments. Asterisks indicate statistical significance versus cells receiving only the vehicle (*p<0.05; **p<0.01). Statistical analysis was performed on ΔCT values with one-way ANOVA. (B) The wild-type *UBC* promoter-pGL3 construct P1 (FR1-2-6) and its mutant counterparts P1mut FR1-2 and P1mut FR6, where HSF binding motifs were selectively mutated within FR1 and FR2 altogether or FR6, respectively, were transfected in HeLa cells. Forty-eight hours post-transfection, cells were treated with the DMSO vehicle or MG132 (20 μM). After 8h, luciferase mRNA levels were determined by RealTime PCR. Expression data, normalized to the housekeeping GAPDH gene, were analyzed by the 2^-ΔΔCT^ method and referred to the value obtained in DMSO-treated cells. The data represent the mean fold induction ± S.E. of 7 independent experiments. Asterisks indicate significant differences of the MG132-induced LUC mRNA fraction between cells transfected with wild-type and mutant reporter constructs (*p<0.05; **p<0.01). Statistical analysis was performed on ΔΔCT values with one-way ANOVA.

To assess the functional role of the *in vitro* identified HSEs we first took advantage of two luciferase reporter constructs present in the laboratory, i.e. P1 and P3 which include both the distal and proximal HSF binding sequences (FR1, FR2 and FR6) or just the proximal one (FR6), respectively (Fig 1). These plasmids were transfected in HeLa cells which were subsequently treated with the proteasome inhibitor MG132 or the vehicle DMSO for 8h. After this time, cells were harvested and RNA was extracted to evaluate luciferase mRNA expression levels by RealTime PCR, using specific primers. Luciferase mRNA levels were measured instead of luciferase activity because treatment with proteasome inhibitors heavily interferes with production of luciferase protein by a post-transcriptional mechanism [34]. Luciferase expression data were referred to those of DMSO-treated cells transfected with the same construct. As shown in Fig 5A, a statistically significant three-fold induction of luciferase expression over the basal level (i.e. DMSO-treated cells) was observed with the P1 reporter plasmid, conversely MG132-inducibility was totally abrogated in the P3 construct lacking the -916/-372 promoter sequence, suggesting that the HSF binding site, identified within the segment FR6 (-100/-65), does not contribute to the transcriptional activation of the *UBC* gene under stress. The role of the HSEs was further investigated by site-directed mutagenesis of the P1 construct where the core GAA sequence contained within the pentameric recognition units, found to be critical for HSF binding *in vitro*, was replaced with the non functional CGC triplet. In particular, two mutated constructs were created: P1 mut FR6, carrying a single substitution within unit 2 of the FR6 segment (FR6 HSFc mut 1X, S5 Fig) and P1 mut FR1-2 where units 1 and 2 were mutated within FR1 (FR1 HSFc mut 2X, S5 Fig) and within FR2 (FR2 HSFa mut 2X, S5 Fig). Mutation of FR1 and FR2 HSF binding motifs altogether (P1 mut FR1-2) almost completely abolished MG132 induction (Fig 5B). By contrast, when the P1 construct was mutated in FR6 (P1 mut FR6) the transcriptional response to MG132 resulted unexpectedly potentiated when compared to that driven by the wild-type P1 construct (Fig 5B). On the whole, the fraction of luciferase mRNA produced in response to MG132 by the P1 mut FR1-2 and by the P1 mut FR6 reporter mutants (Fig 5B) was significantly reduced (70%) and increased (150%), respectively, compared to P1, as demonstrated by the statistical analysis of the ΔΔCt values.

## Discussion

Despite the fact that several types of stress are able to induce ubiquitin expression in mammals [35], little is known about the molecular players involved at the DNA level. The polyubiquitin genes *UBB* and *UBC* are stress-regulated genes containing putative heat shock elements in their promoter regions [9,15]. In agreement with this observation, genome wide ChIP-Seq analysis has recently revealed that both HSF1 and HSF2 bind to their promoter regions after heat-shock treatment, but only depletion of HSF1 seems to compromise the heat-induced ubiquitin expression [18]. Far less is known about the molecular architecture and the activity of the *cis*-acting elements mediating Ub gene induction under proteotoxic stress. We have recently demonstrated that in HeLa cells only the polyubiquitin genes *UBB* and *UBC* are up-regulated upon proteasome inhibition, oxidative stress and heat shock with markedly higher responses from the *UBC* promoter [36]. In this paper, the *UBC* promoter region has been dissected at the molecular level to map and functionally characterize its heat shock elements under conditions of proteasome inhibition by MG132. Differently from other proteotoxic stressors, proteasome inhibitors activate all members of the heat shock family, including HSF2 [37]. Indeed, HSF2 is a short-lived protein whose activation involves protein accumulation as the consequence of an increased synthesis and/or decreased degradation by the proteasome [32]. In addition, based on the evidence that proteasome inhibitors are in clinical use to treat certain types of cancer, the effects produced by these molecules at the genomic and proteomic level are of interest for improving their therapeutic properties [38]. In agreement with the evidence that different classes of proteasome inhibitors are able to activate HSFs and induce the heat shock response [32,37], HSF occupancy at the *UBC* promoter regions FR1 and FR6 was also demonstrated in cells treated with lactacystin, a more specific and irreversible inhibitor molecule. After 4h of treatment, the response was quantitatively lower than in cells incubated with MG132. This evidence could be more likely explained by a different kinetic of HSF activation rather than a different mechanism of action of the two inhibitors. Indeed, in response to lactacystin treatment, the same extent of HSFs binding was also detected on the *Hsp70* promoter, a well characterized HSF-responsive DNA element.

By using *in silico* and *in vitro* approaches three *UBC* promoter regions recruiting HSF1 and HSF2 were identified: two distal (nt -841/-817 and nt -715/-691) and one proximal (nt -100/-65) to the transcription start site. Notably, all these regions are comprised within the HSF1/HSF2 target sites previously revealed by ChIP-Seq analysis, although a different cell line (i.e. K562) and a different stressor (i.e. heat shock) were investigated [18]. In particular, for HSF1 two target sequences were reported which include the two distal and the proximal site(s), respectively. The point with the highest HSF enrichment is located between FR1 and FR2 (-752), in the first case, and in very close proximity to FR6 (-58), in the second case. By contrast, only one target site of 1800 bp was found for HSF2 with a ChIP peak summit at -863 that is immediately upstream of the FR1 HSE we have identified. Since, as explained above, HSF2 is activated by proteasome inhibition and not by heat shock, the binding profile of HSF2 at the *UBC* promoter could be qualitatively and quantitatively very different, explaining the differences between the results presented in this study compared to those obtained by Vihervaara et al. [18].

It is well known that HSFs regulate the expression of target genes via heat shock elements which consist of the minimum sequence nGAAnnTTCnnGAAn [39]. Although the exact identification of nucleotides composing the HSE motifs is beyond the scope of this paper, the data presented do allow some comments upon the structure of the potential *UBC* HSEs within each mapped stress-responsive sequence. However, since HSEs are very plastic elements and the following observations are mainly based on *in silico* results, which only predict the presence of an HSF binding site, it is worth noting that in most cases they can be considered speculative. In ODN FR1 HSFc (-841/-817) the two GAA blocks, retrieved by computational analysis, together with the nucleotide ^-831^GTC^-829^ triplet, laying in between, may constitute the following HSE consensus sequence ^-837^A**GAA**CA**GTC**TA**GAA**G^-823^, which resembles the canonical one, with the exception that the T is replaced by G within the TTC block. Indeed, beside guanines that are the most conserved nucleotides, substitutions in the sequence of the trinucleotide blocks are tolerated in an active HSE regulatory element [40]. In addition, it has been demonstrated that HSEs still function if they contain one [gap-type, nTTCnnGAAn(5 bp)nGAAn] or two [step-type, nTTCn(5 bp)nTTCn(5 bp)nTTCn] gaps between the units [16]. A typical discontinuous gap-type HSE is that present in ODN FR2 HSFa (-715/-691) where the first ^-693^nGAAn^-697^ motif, in antisense orientation, is spaced of 5 bp (in brackets) from the second one: ^-712^G**GAA**AG**TTC**T(CGAGC)G**TTC**C^-693^. Conversely, ODN FR6 HSFc (-100/-65) appears to contain an atypical step-type HSE element where the first ^-69^nGAAn^-73^ unit, in antisense orientation, is spaced from the second ^-79^nGAAn^-83^ of 5 bp, while a putative third ^-92^nTCCn^-96^ motif exists 8 bp downstream of the latter: ^-96^G**GAA**A(AGTAGTCC)C**TTC**T(CGGCG)A**TTC**T^-69^. Gaps between the second and third units longer than 5 bp have been previously reported, such as that of 11 bp described in the *Saccharomyces cerevisiae MDJ1* gene; the authors demonstrate that this HSE is still functional [41]. Because each DNA binding domain of a trimeric HSF binds to the target sequence, the typical HSE contains at least three nGAAn units. However, HSEs with more than three nGAAn motifs exist which bind HSF trimers cooperatively. High affinity binding sites for HSF were found to consist of multiple inverted repeats of the nGAAn motif within the promoter of heat shock proteins [42]. In the case of the *UBC* promoter it cannot be excluded that the HSEs identified within FR1 and FR6 HSFc ODNs may include *in vivo* additional GAA blocks. Indeed, putative nGAAn motifs were also found in the 5’ flanking oligonucleotides (i.e. FR1 HSFb and FR6 HSFb), but these sequences showed a scarce ability to bind HSFs *in vitro*. Thus, it could be speculated that they may contain a part of the downstream HSE which has been physically separated from the rest because of the fractionation strategy employed.

Functional characterization of the *UBC* HSEs, by using 5’-deleted and mutated reporter constructs, has clearly demonstrated that the distal regions contained within ODN FR1 HSFc (-841/-817) and FR2 HSFa (-715/-691) are responsible for the up-regulation of *UBC* transcription upon MG132 treatment; by contrast, the proximal (-100/-65, ODN FR6 HSFc) is per se not responsive to stress (construct P3). This evidence suggests that this HSE could be not functional. Surprisingly, when it was mutated to disrupt one of the *in vitro* identified HSF recognition units (P1 mut FR6), the transcriptional activity driven by the distal stress-responsive elements significantly increased, suggesting that, in the context of the *UBC* promoter, this atypical step-type heat shock element may have a regulatory repressive function. It has been demonstrated that gap-type HSEs sustain moderate stress-induced transcription, while step-type HSEs mediate low level activation [21]. Thus, to the best of our knowledge, this is the first report of an HSE with a negative role on the stress-induced transcriptional activity of a gene promoter. It could be hypothesized that the -100/-65 HSE is able to specifically recruit HSF family members which can exert a repressive activity. Indeed, there is evidence that HSF1 preferentially binds canonical HSEs such as that contained in the distal FR1 *UBC* promoter region, while HSF2 preferentially interacts with discontinuous HSEs such as that detected in the proximal FR6 segment [43]. In addition, it has been demonstrated that, during heat shock or MG132 treatment, HSF2 is able to repress expression in a gene-specific manner, probably by modulating HSF1 activity [17]. This ability has been attributed to the formation of heterotrimers, but their exact role is not yet fully understood [19]. In particular, it is not clear how HSF2-containing heterotrimers can have opposite effects on the transcriptional activity of different genes, activating in one case and repressing in another. The results obtained by ChIP assay clearly indicate that both HSF1 and HSF2 bind to the distal and proximal HSEs of the *UBC* promoter in basal conditions and after proteasome inhibition. Notably, under stress conditions, the fold-induction of HSF1 over its basal level was much greater than that of HSF2 in the distal HSEs (FR1), as it occurs on the *HSP70* promoter. By contrast, a similar extent of induction of HSF1 and HSF2 factors was observed in the proximal HSE (FR6). Thus, it could be speculated that, upon proteotoxic stress, HSF1 transactivating homocomplexes may be preferentially recruited to the distal elements, while HSF1/HSF2 heterocomplexes with repressing function are probably assembled on the proximal HSE. In addition, HSF2 exhibits two alternative splicing isoforms, at least in mice, one of which is able to reduce HSF1 activity by forming heterocomplexes with the HSF1β isoform under MG132 treatment [20]. Unfortunately, to date, the lack of information on HSF isoforms in humans and the unavailability of specific antibodies, render difficult to test their involvement. HSF heterotrimer formation and changes in the expression pattern of HSF isoforms in a cell- and stress-type manner are certainly important mechanisms regulating the level of the heat shock response. However, to the state of the art, they do not allow to envisage a possible scenario explaining how heat shock factors binding to one HSE may negatively modulate the transcriptional activity driven by HSFs bound to HSEs located 600–700 bp upstream of the former, on the same gene promoter. Thus, the repressive mechanism of action may be, more likely, searched in a co-factor(s) which is specifically recruited by different HSF family members, isoforms or trimers, interacting with specific HSE configurations, and in response to different stimuli. Besides activators/co-activators and co-repressors, some repressor proteins may directly target basal transcription factors or RNA polymerase itself, thus hindering, in some manner, the assembly of the transcriptional apparatus. In the case of HSF family members, it has been demonstrated that the splice variant HSF-4a represses transcription at an early step during preinitiation complex assembly via interaction with the basal transcription factor TFIIF [44]. HSF4 is not expressed in HeLa cells, but it could be hypothesized that other HSF isoforms with repressive functions may act trough a similar mechanism. The closed proximity of the -100/-65 HSE to the transcription start site suggests that the factor(s) recruited may easily contact the basal transcription complex and interfere with its assembly and/or activity.

In conclusion, data reported in this paper demonstrate, for the first time, that the presence of multiple HSEs, spread over several hundred base pairs and with multiple configurations, can create a stress-responsive architecture where DNA regulatory regions with activating and repressor function could coexist. At the moment, the mechanism through which repressive elements may modulate the activity of canonical HSEs, which function as activators, is not known. To date, the information regarding HSF *trans*-acting factors, on the one hand, and the HSE *cis*-acting elements, on the other, are not yet sufficiently integrated to allow to fully understand their interaction dynamics. The results presented in this paper open new questions on how the stress response is orchestrated and fine-tuned at the DNA level, highlighting a new layer of regulation of HSF activity. In addition, they further clarify the mechanisms regulating *UBC* expression, which have important implications both for the understanding of how ubiquitin levels are controlled during the stress response and for the designing of vectors for gene therapy. Indeed, the *UBC* promoter is already used to drive expression of transgenes in mammals [45,46]. Thus, the possibility to improve/modulate *UBC* performance by engineering its regulatory sequences is of particular interest.

## Supporting Information

S1 Fig Time course analysis of HSF activation following MG132 treatment. Nuclear extracts, obtained from cells treated with MG132 for different period of time, were incubated with a ^32^P-labeled ODN containing the canonical HSF consensus sequence (ODNc-HSE).
(TIF)

S2 Fig EMSA competition experiments to identify *trans*-acting protein factors binding to FR1-FR6 *UBC* fragments. Nuclear extracts, obtained from cells treated with MG132, were pre-incubated with an ODN containing the wild-type (WT) or mutated (MUT) Sp1 or YY1 consensus sequence prior to the addition of the ^32^P-labeled FR1, FR2, FR3 (panel A), FR4, FR5 and FR6 (panel B) probes. ODN sequences can be found in [23,31].
(TIF)

S3 Fig ChIP analysis of Lactacystin-treated cells. ChIP analysis was performed on DMSO- and Lactacystin-treated cells (10 μM, 4h) using specific antibodies against HSF1 and HSF2. Each sample was tested in triplicate. The average value ± SE was calculated from two independent ChIP analyses.
(TIF)

S4 Fig DNA sequences of the *UBC* FR1, FR2 and FR6 segments and of the selected ODNs. The GAA core of the putative pentameric HSE recognition units is highlighted in boldface. Arrows indicate strand orientation. Synthetic ODNs (nucleotides in black within boxes) were designed in order to include at least two closed nGAAn recognition motifs. ODN name and position (in brackets) with respect to the transcription start site (+1) are reported.
(TIF)

S5 Fig Sequences of mutant ODNs. The core GAA (in boldface, underlined) within the nGAAn putative recognition unit was substituted with the non-functional CGC triplet (in boldface, marked in gray). A number (within black dots) has been arbitrarily assigned to each nGAAn motif in order to facilitate the description.
(TIF)
