# Supplementary figures and images for: Molecular Dissection of the Human Ubiquitin C Promoter Reveals Heat Shock Element Architectures with Activating and Repressive Functions
Source: PLoS One. 2015 Aug 28;10(8):e0136882. doi: 10.1371/journal.pone.0136882 (PMC4552642; doi:10.1371/journal.pone.0136882)

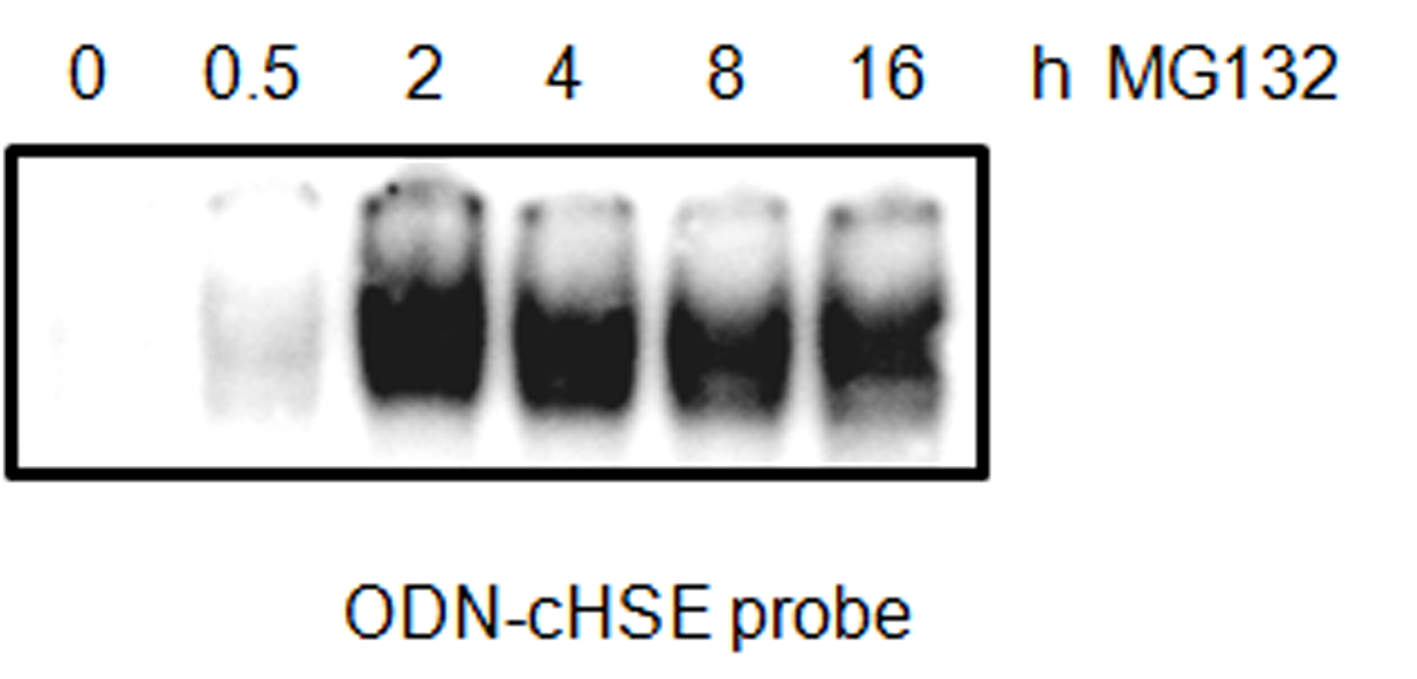

Supplement: S1 Fig — Nuclear extracts, obtained from cells treated with MG132 for different period of time, were incubated with a 32P-labeled ODN containing the canonical HSF consensus sequence (ODNc-HSE). (TIF) [file pone.0136882.s001.tif]

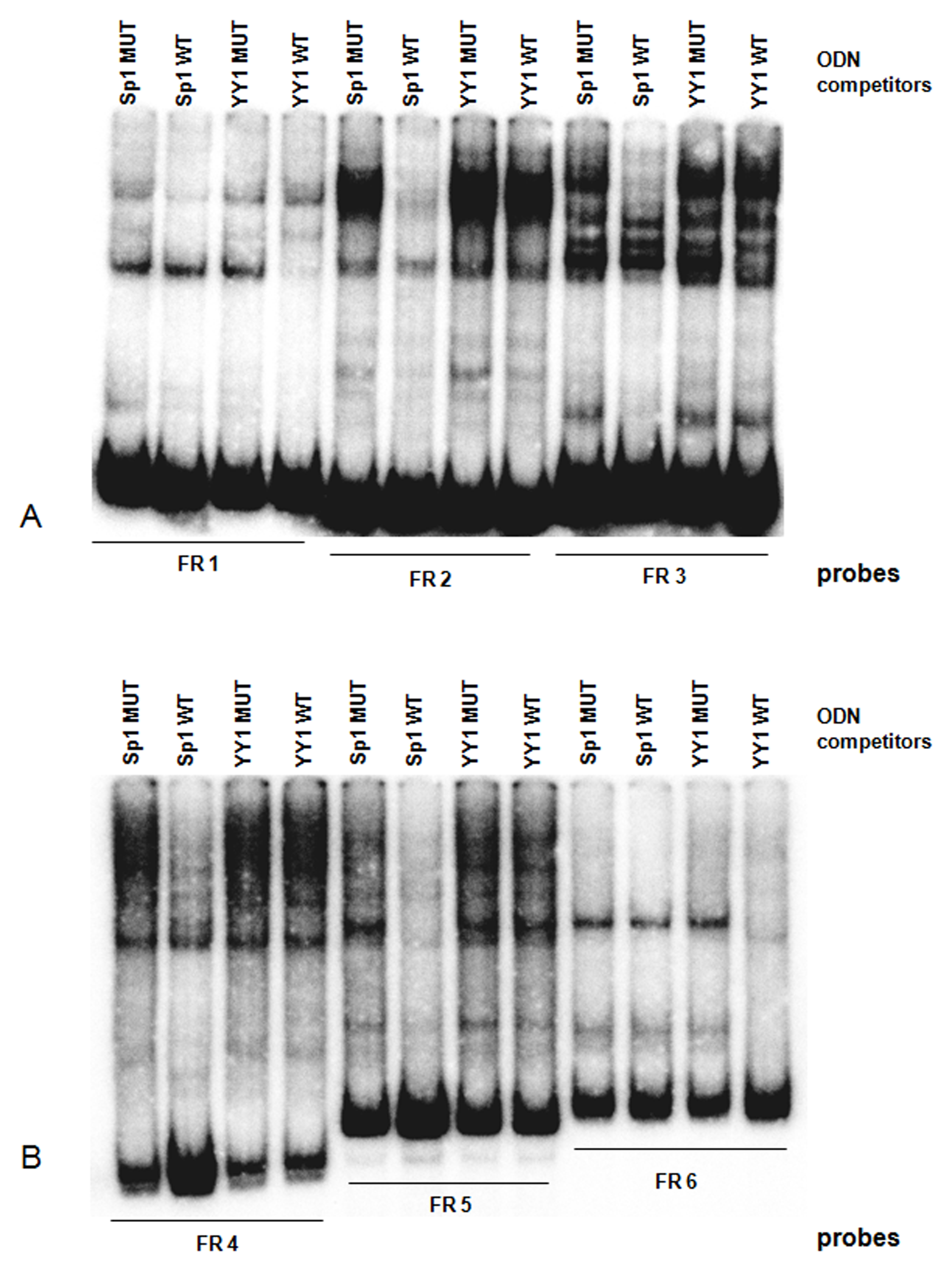

Supplement: S2 Fig — Nuclear extracts, obtained from cells treated with MG132, were pre-incubated with an ODN containing the wild-type (WT) or mutated (MUT) Sp1 or YY1 consensus sequence prior to the addition of the 32P-labeled FR1, FR2, FR3 (panel A), FR4, FR5 and FR6 (panel B) probes. ODN sequences can be found in [23,31]. (TIF) [file pone.0136882.s002.tif]

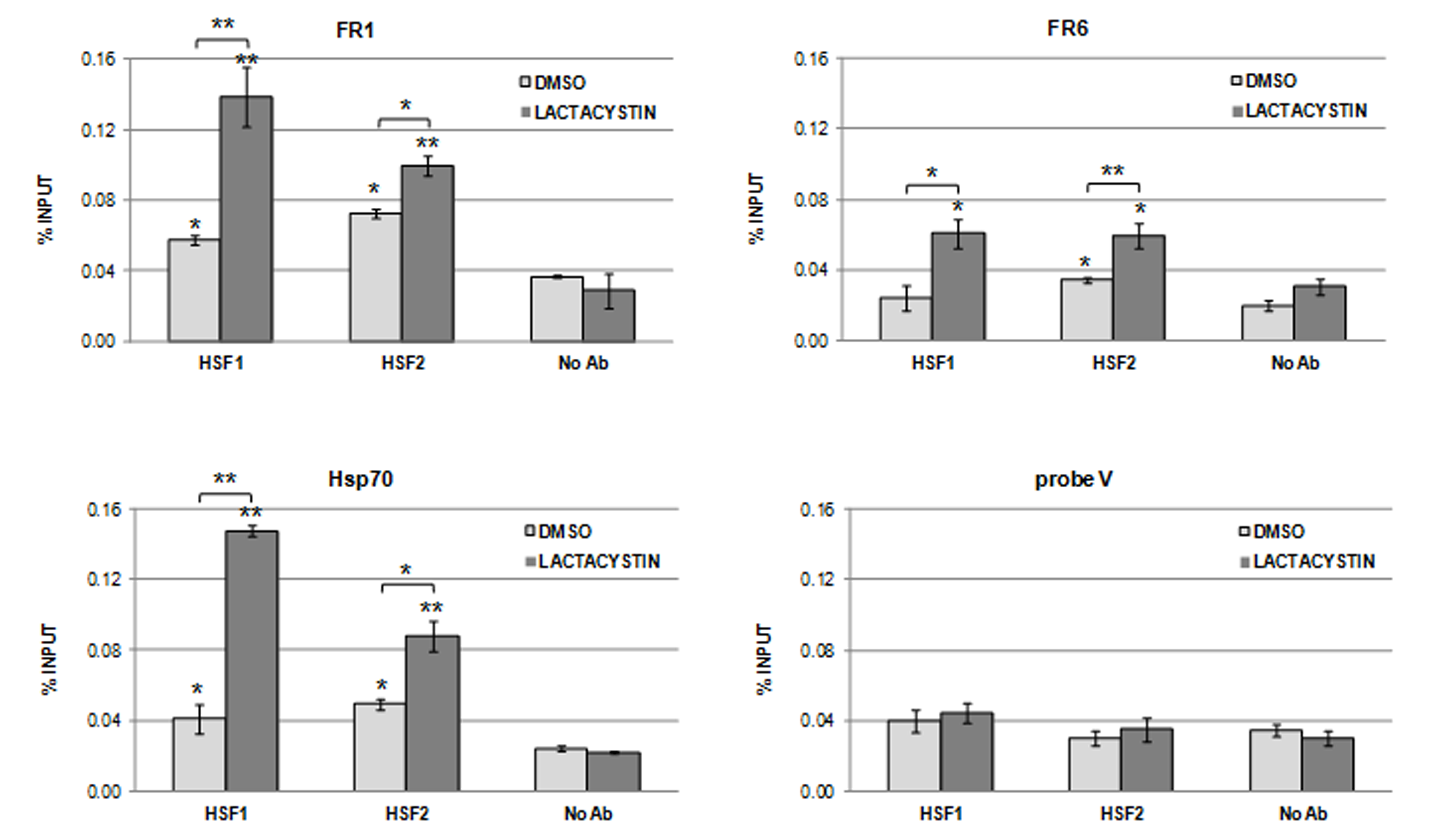

Supplement: S3 Fig — ChIP analysis was performed on DMSO- and Lactacystin-treated cells (10 μM, 4h) using specific antibodies against HSF1 and HSF2. Each sample was tested in triplicate. The average value ± SE was calculated from two independent ChIP analyses. (TIF) [file pone.0136882.s003.tif]

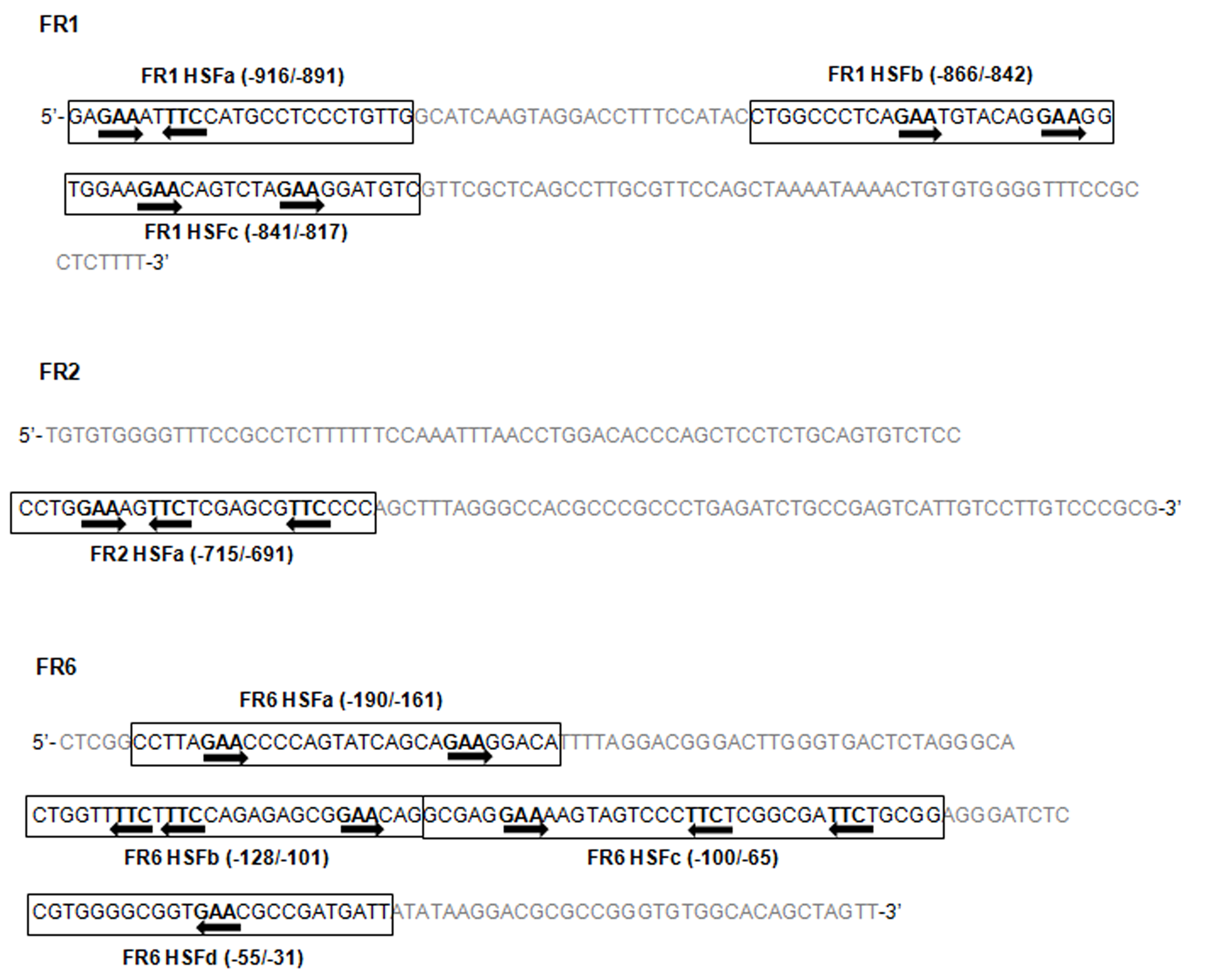

Supplement: S4 Fig — The GAA core of the putative pentameric HSE recognition units is highlighted in boldface. Arrows indicate strand orientation. Synthetic ODNs (nucleotides in black within boxes) were designed in order to include at least two closed nGAAn recognition motifs. ODN name and position (in brackets) with respect to the transcription start site (+1) are reported. (TIF) [file pone.0136882.s004.tif]

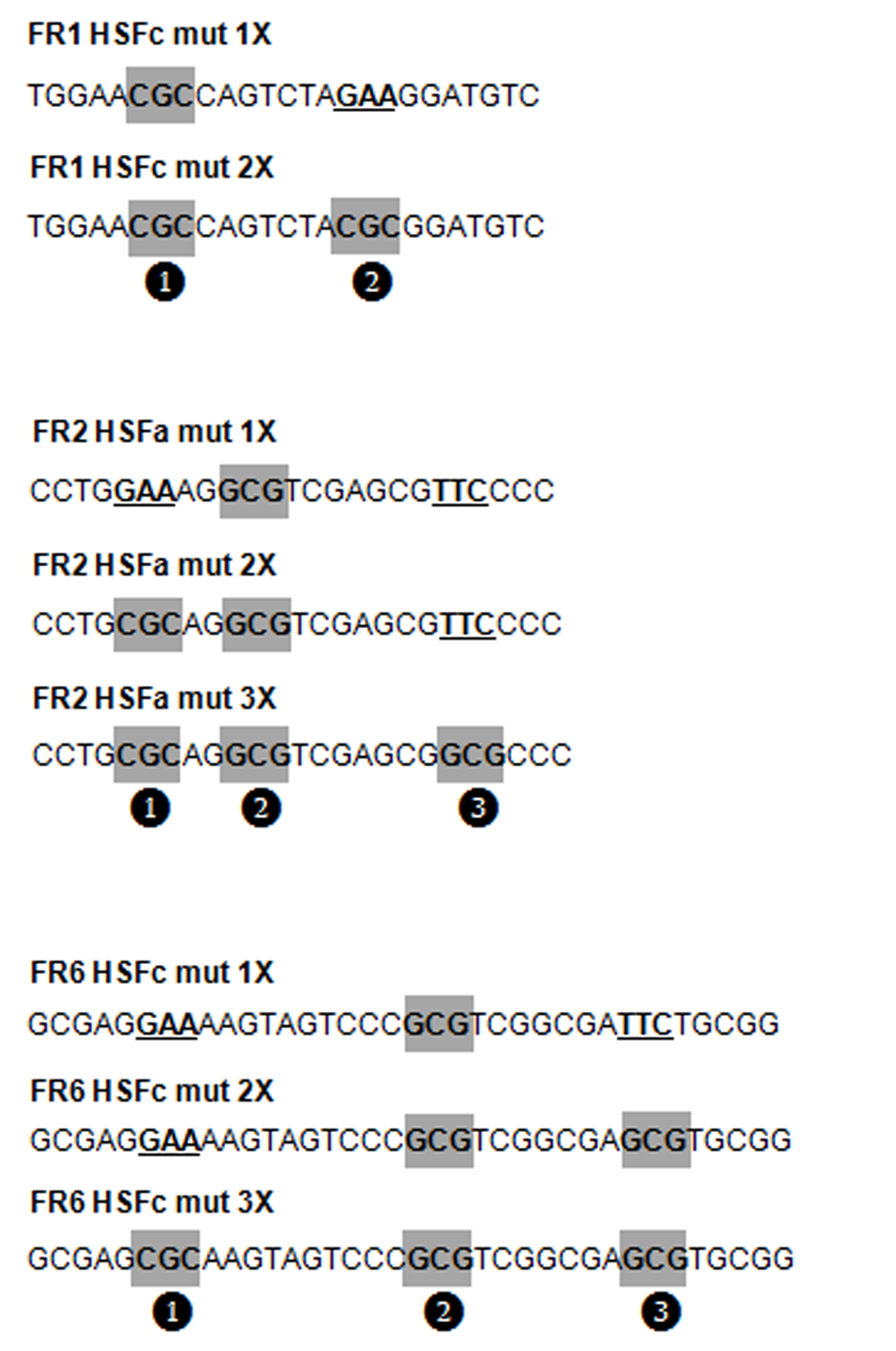

Supplement: S5 Fig — The core GAA (in boldface, underlined) within the nGAAn putative recognition unit was substituted with the non-functional CGC triplet (in boldface, marked in gray). A number (within black dots) has been arbitrarily assigned to each nGAAn motif in order to facilitate the description. (TIF) [file pone.0136882.s005.tif]
